# The Modern Treatment of Tuberculosis of the Spine

**Published:** 1920-03

**Authors:** A. Rendle Short

**Affiliations:** Assistant Surgeon to the Bristol Royal Infirmary


### THE MODERN TREATMENT OF TUBERCULOSIS OF THE SPINE.

BY

A. Rendle Short, M.D., B.S., B.Sc., F.R.C.S.,

*Assistant Surgeon to the Bristol Royal Infirmary.*

---

Until the past few years tuberculous caries of the spine has been one of the most tedious of diseases. There are considerable risks to life. According to some old authorities, about a third of the cases used to die within a few years of the onset. More recent figures are much more favourable in this respect, but the true mortality has still been as high as 5 or 10 per cent. Tubby followed ninety-nine cases for two and a half years, and of these twelve died. Even those who survive are apt to be delicate all their days.

In private practice, or at a special hospital such as Alton, better results can be obtained, and between 80 or 90 per cent. of those in whom treatment is undertaken early, in the pre-curvature stage, are likely under best conditions to make a good recovery. If town dwellers of the hospital class are included, the results are not nearly so good; probably in the past not more than half reach a stage of permanent comfort. In adults, particularly, it has been very unusual to see lasting good results.

The principal drawback of the disease, however, has been the tedious nature of its treatment. It is necessary that the patient should be kept absolutely recumbent, without once sitting up, for a prolonged period—six months for the cervical spine, eighteen months for the dorsal, and twelve months for the dorsi-lumbar (Fraser). Even these times are not enough to ensure safety in the case of adults. After that some form of apparatus must be worn, probably for two years, and sometimes for longer. Seeing that at the end of this time the sufferer will probably be hunch-backed from long slow collapse of the centra of the vertebræ, it is not surprising that a tuberculous spine is regarded with feelings akin to despair by the patient and relatives.

One of the few blessings brought us by the war was the opportunity to meet and converse freely with some of the best American surgeons, who had come over to France and were temporarily attached to British hospitals there. One could therefore profit by the experience, not only of the brilliant and sometimes too sanguine inventors of new treatments, but also of the more cautious rank and file. I was greatly impressed by the glowing terms in which a number of them spoke of Albee's operation for tuberculous caries of the spine. One of them described it as " one of the most gratifying operations in surgery." I was therefore encouraged to make trial of the method, and so far have operated on five patients. Of these the fifth is still in hospital, and although he is doing well I shall not describe the case in detail.

The technique, very briefly, is as follows. By means of an electric cable saw, or an Albee saw, a graft about five or six inches long is cut from the subcutaneous surface of the tibia. If the crest is spared the bone is not weakened. Then the spines of five vertebræ, including the most prominent spine at the apex of the angular curvature, two above and two below, are split, not quite in the middle line, by the chisel, and a bed made for the graft to lie in. It is necessary sometimes to include a sixth spine. The graft is fixed by catgut sutures, uniting its periosteum with that of the fasciæ of the back, and as far as possible these fasciæ are brought over the graft to bury it.

In one of my cases it was necessary to bend the graft on account of the severity of the angular curvature. This bending is facilitated by four or five cross-cuts with the saw on the marrow surface of the graft, going half-way through it. The operation should not involve any particular degree of shock or bleeding.

The patient is kept absolutely recumbent on a bed with a pulley arrangement for raising him without rolling or sitting him up. At the end of two months a light plaster jacket is fitted, which is to be worn for about six months. As soon as the jacket is firmly set and dry the patient is allowed up.

As will be seen by the four case-reports appended, three patients have been restored by this treatment to a condition which, so far at any rate, may be described as a cure; they are able to get about freely and without pain, and there has been no increase of deformity beyond that which they had developed at the time of the operation. The fourth case is undoubtedly a great deal improved. Two of the four patients had been treated for years by other methods without success.

*Indications and Contra-indications.*—In view of the small risks of the operation and its many advantages, I am of opinion that it should be the routine treatment of all cases of tuberculous spine, unless contra-indications are present. These advantages are: (1) The great shortening in the time to be spent in bed—the period is reduced from twelve or eighteen months to two months; (2) the shortening of the time during which an apparatus or jacket must be worn; (3) the increased proportion of the cases who attain to a life of comfort, at any rate in patients of the hospital class—it is particularly amongst adults that this advantage is seen, because in them it is so difficult to obtain a lasting cure by recumbency; (4) the avoidance of any increase in the deformity.

The principal contra-indications are as follows: In children under the age of twelve the vertebral spines are so slender that it is difficult to split them properly, and the operation may fail. In the cervical region, at any age, the same difficulty prevails. If a dorsal abscess is present the graft might not unite. I have refused one case because there were two tuberculous foci in the spine, another because several bones and joints were also affected, and a third because paraplegia was present; but these are relative rather than absolute contra-indications. Albee himself recognises no contra-indications except sepsis.

*Rationale of the Treatment.*—The main agency by which healing may be obtained in a tuberculous bone is rest. When the disease is situated in the centra of one or more vertebræ this rest may be obtained effectually by prolonged and absolute recumbency, but when the patient gets about again the more or less perfectly recovered area is bound to be subjected to strain and pressure. Obviously such external support as may be afforded by a jacket, though it may protect against movements, cannot help much in taking off the pressure.

When the graft is introduced, and has become welded by firm bony union to the two vertebral spines above the apex of the angular deformity and to the two below, the five spines form one rigid mass quite capable of acting as an efficient splint. Aided by the articular processes and pedicles of the vertebræ, it can transmit the weight of the upper part of the body without involving the centra in any injurious pressure. They are further protected from such a degree of collapse as would exaggerate the hunch-back deformity.

Non-union of the graft seldom, if ever, occurs unless there is gross suppuration.

*Treatment of Spinal Caries apart from Operation.*—Some improvements have been made of late in the appliances which may be used both during the period of recumbency and when the patient is up and about.

For the first period probably nothing is better than a Phelps box for young children and a spinal carriage for older children and adults. Where this latter cannot be obtained the Whitman frame presents great advantages. It consists of an oblong made of half-inch gas-piping, as wide as the patient's shoulders and eight inches longer than his height; over this frame canvas is stretched for him to lie upon, with a mackintosh beneath the buttocks. A canvas apron is tied over the patient to keep him from rolling off or sitting up. The advantages of this simple contrivance are that it can either lie on the bed or be carried into the garden, and that by bending the frame opposite the angular deformity some protection may be given against its increase.

When at the end of twelve or eighteen months the patient begins to get up he is apt to faint. Sir H. Gauvain has invented a tip-up carriage to be used at this stage, to accustom the sufferer to the semi-erect attitude by degrees before he leaves his couch.

When the period of recumbency has passed, and the time for getting about has arrived, the question of the most suitable spinal support arises. A well-applied plaster jacket, fitting snugly over the great trochanters, is efficient but very heavy. Better is Gauvain's celluloid substitute, impregnated with calcium chloride to render it non-inflammable. It is much lighter to wear, but it is a long, tedious business for the doctor or the nurse to prepare it.

Poroplastic and leather jackets save the doctor trouble, but are very little support for the patient's spine. A well-made Taylor brace, with a pelvic band and two paravertebral rods with a jacket fitted to them, is better. One or other of these supports should be worn for two years, and longer still if pain returns.

Cases of cervical caries are not very suitable for the Albee bone-graft, but fortunately they do not require so long a period of recumbency. Six months will usually suffice. During this time some means must be found to fix the head, either a weight extension or sandbags on either side. Only one very small pillow must be allowed.

When the patient gets up the best appliance is a plaster or celluloid jacket, either of the "Minerva" or the "fillet" type. In the "Minerva" the jacket fits over the trochanters and covers the abdomen, chest and shoulders, with arm-holes and an abdominal window; it then embraces the neck and takes the weight of the head, reaching as high up as a line drawn from the external occipital protuberance just below the ears to the point of the chin. This fixes the lower jaw and is awkward for eating. The "fillet" improves upon it by cutting away a piece under the chin sufficiently to free the jaw, and to compensate for this a band of plaster is carried from the external occipital protuberance around the forehead. These appliances are effective but not comfortable, and after six months may be replaced by a jury-mast attached to an ordinary plaster jacket, or a Taylor ring to support the occiput and chin attached to a Taylor brace.

*Four Cases treated by Albee's Operation.*

CASE 1.—H. H., boy, aged 12. Four months' history. For three months unable to walk on account of pain in the back and legs giving way. No actual paraplegia. Angular deformity, slight, in lower dorsal region. Skiagram shows blurring. Operation May 14th, 1918. Was running about in three months in comfort. At Christmas, 1919, was visiting the Infirmary free from pain and able to do anything.

CASE 2.—C. H., man, aged 36. Trouble dates from blow on back in 1914. Has been treated as out-patient and in-patient at Guy's and St. Thomas's, with rest in bed for long periods, and wore a poroplastic jacket. Still great pain and weakness. Marked angular deformity in lower dorsal region. Operation

October 15th, 1918. Was getting about without pain in three months, and greatly regretted that he had not had the operation long before. Seen by Dr. L. J. Short in January, 1920. Patient is of a grumbling type, and has phthisis. His back is firm and free from all but slight pain, and he can get about the house.

Case 3.—C. M., man, aged 32. Seven years' history. In 1916 was treated by a year in bed, then got up with plaster jacket, followed by poroplastic jacket. Pain returned in 1918; by spring of 1919 was quite incapacitated, and cannot sit up without the jacket on. Angular deformity over 10th dorsal spine, moderate degree. Operation July 1st, 1919. Is now able to walk several miles with little or no pain, and has returned to work (as a tailor).

Case 4.—S. C., man, aged 33. Slight symptoms for eighteen months, severe pain and disability for two months. Cannot walk more than a few yards. Angular deformity over 8th dorsal spine. Operation July 29th, 1919. Seen in February, 1920. Can walk ten miles, and is quite free from pain. Wearing a light plaster jacket still.

### REFERENCE.

Albee, *Surg. Gynæ. and Obstet.*, 1914, vol. xviii. p. 699.

---

